# The Associations between Dietary Patterns and Sedentary Behaviors in Polish Adults (LifeStyle Study)

**DOI:** 10.3390/nu10081004

**Published:** 2018-08-01

**Authors:** Marzena Jezewska-Zychowicz, Jerzy Gębski, Dominika Guzek, Monika Świątkowska, Dagmara Stangierska, Marta Plichta, Milena Wasilewska

**Affiliations:** 1 Department of Organization and Consumption Economics, Faculty of Human Nutrition and Consumer Sciences, Warsaw University of Life Sciences (SGGW-WULS), 159C Nowoursynowska Street, 02-787 Warsaw, Poland; jerzy_gebski@sggw.pl (J.G.); dominika_guzek@sggw.pl (D.G.); monika_swiatkowska@sggw.pl (M.Ś.); marta_plichta@sggw.pl (M.P.); milena_wasilewska@wp.pl (M.W.); 2 Section of Horticultural Economic, Faculty of Horticulture, Biotechnology and Landscape Architecture, Warsaw University of Life Sciences (SGGW-WULS), 159C Nowoursynowska Street, 02-787 Warsaw, Poland; dagmara_stangierska@sggw.pl

**Keywords:** dietary patterns, physical activity, sedentary behaviors, adults, principal component analysis

## Abstract

Sedentary behavior, a low physical activity level, and unhealthy dietary patterns are risk factors for major chronic diseases, including obesity. The aim of this study was to assess the associations of dietary patterns (DPs) with sedentary behaviors (SB) and self-reported physical activity (PA). The data was collected in November 2016 through a cross-sectional quantitative survey amongst 1007 Polish adults. Principal components analysis (PCA) was conducted to derive DPs. Logistic regression analysis was used to verify associations between PA and SD (independent variables), and DPs (dependent variables). Five DPs (‘Fast foods & sweets’—FF&S; ‘Meat & meat products’—M&MP; ‘Fruit & vegetable’—F&V; ‘Wholemeal food’—WF; ‘Fruit & vegetable juices’—F&VJ) were identified. Representing M&MP independently increased the chance of watching TV at least once a day (by 73%). There was no such relationship between the FF&S and sedentary behaviors. Being in the upper tertiles of pro-health DPs increased the chance of reading books (by 177%—F&V, 149%—WF, 54%—F&VJ) and watching TV (by 71%—F&V). On the other hand, belonging to the upper tertile of WF reduced the chance of using the computer for more than 4 h a day. Belonging to the upper tertile of healthy DPs (WF and F&VJ) increased the chances of moderate or high physical activity, both at work/school and during leisure time. Within F&V, there was a lower chance of moderate or high physical activity at work/school. Being in the upper tertile of unhealthy DPs (FF&S and M&MP) did not show any significant association with physical activity. The study indicated the associations between both healthy and unhealthy DPs and some sedentary behaviors. Association between F&V and watching TV and reading books/newspapers should be recognized as potentially efficient in education. Association between M&MP and watching television can be indicative of the mutual overlap of a negative lifestyle resulting in the development of overweight and obesity, especially since the extent of occurrence of sedentary behaviors is high.

## 1. Introduction

Sedentary behavior and a low physical activity level are risk factors for major chronic diseases such as obesity and type 2 diabetes, cardiometabolic risk, and mortality [1,2,3,4]. In contrast, regular physical activity and fitness has been shown to improve health and decrease the risk of chronic diseases, frailty, disability, falls, and mortality [5,6,7]. Simultaneously sedentary behaviors that involve activities with a very low energy expenditure (1.0–1.8 metabolic equivalents), performed mainly in a sitting or supine position [8], were found to be associated with the consumption of unhealthy foods in youth [9,10] and in adults [11,12,13], and were linked to an increase in obesity as well as to the higher probability of developing chronic diseases [1,14,15].

In previous studies, watching television time has been used most often as a measure of sedentary behavior [12,16]. The studies have found associations between time spent watching TV and obesity, cardiovascular risk factors, metabolic syndrome, and type 2 diabetes [2,17]. Few studies have evaluated whether dietary intakes may mediate the effects of watching TV on cardio-metabolic risk factors [3,18,19,20]. Watching TV has been shown to coincide with extra calorie intake during this activity [21,22,23]. While watching TV, one is exposed to an increasing number of advertisements for food rich in fat, sugar, and salt [24,25], which promotes the consumption of these products [21,26,27]. 

In nutritional epidemiology, dietary patterns (DPs) have been attracting a lot of attention in the last decades [28,29,30,31]. The studies have shown that DPs are significantly associated with cardiovascular disease, overweight and obesity, and other diseases, e.g., cancer, Alzheimer disease, and mild cognitive impairment [32,33,34,35], and with other elements of individuals’ lifestyle, e.g., physical activity [36,37,38,39]. Nevertheless, the associations between dietary patterns and physical activity are still unclear [40]. There is a need to identify associations between them and potentially modifiable components, e.g., particular sedentary behaviors. Research methods used in investigating the association between dietary habits and physical activity found in previous studies have certain limitations. A number of studies have examined these associations in relation to the consumption of particular foods (i.e., fruits, vegetables, high-fat foods), indicating that people with higher physical activity are more likely to make healthier dietary choices than those with lower physical activity [9,41,42,43]. However, some studies demonstrate that a higher level of physical activity may not counteract an unhealthy diet [44].

The available evidence for the existence of associations between dietary habits, sedentary behaviors, and physical activity is quite strong for children and adolescents [45,46,47], but still limited for adults [37,38]. Moreover, previous studies focused solely on television viewing, which is only one domain of sedentary behavior. Other domains have to date been rarely associated with dietary patterns reflecting the complexity of dietary behaviors [48]. In contrast to particular dietary characteristics, that is, foods and/or nutrients, DPs represent the whole diet [49]. Knowing the associations between DPs and domain-specific sedentary behaviors is needed to better define a healthy lifestyle and its determinants and target at-risk groups in education programs. To address the gaps in the evidence, the aim of this study was to assess the association of dietary patterns with sedentary behaviors and physical activity, derived from a self-reported questionnaire carried out in a representative sample of the Polish population. The diagnosis of this relationship is justified from the perspective of disease prevention.

## 2. Materials and Methods

### 2.1. Ethical Approval

The study was approved by the Ethics Committee of the Faculty of Human Nutrition and Consumer Science, Warsaw University of Life Sciences, in Poland, on the 27th of June 2016, (Resolution No. 01/2016). Informed consent to participate in the study was collected from participants.

### 2.2. Study Design and Sample Collection

The data was collected in November 2016 through a cross-sectional quantitative survey under the LifeStyle Study. According to the study design, recruitment and data collection were conducted by a research agency—ARC Market and Opinion. Participants were selected from the panel of approximately 55,000 registered adult people. Members of epanel.pl received monetary gratification for participation in the study. In order to obtain remuneration, epanel.pl participants had to fill out the payout form, in which they gave their name, PESEL, and address of check-in. Only people aged 21–65 qualified for the study.

After sending an invitation to participate in the LifeStyle Study, 6910 people clicked on the link, thus expressing their willingness to participate in the survey. Quota selection using gender, age, place of residence, and region was used to ensure the representativeness of the Polish population. During the recruitment for the study, five people did not meet the panel criteria (age above 65), 144 people stopped filling out the questionnaire during the interview, and 5746 people did not qualify due to filling the quota, while eight people were removed from the database at the collection control stage due to very short time of completing the questionnaire and the same answers to all questions on the frequency of eating. As a result, the study consisted of 1007 participants. The computer-assisted web interviewing (CAWI) technique was used to collect all data.

### 2.3. Eating Habits

The Beliefs and Eating Habits Questionnaire (KomPAN) developed and validated by the Commission of Behavioral Determinants of Nutrition from the Polish Academy of Sciences (Warsaw, Poland) [50] was used to assess the frequency of the consumption of selected food groups, including: whole meal bread; whole meal pasta and groats; fermented milk drinks; cheeses (including melted cheese, blue cheese); cured meats and sausages; red meat; white meat; fried foods; fruits; vegetables; vegetable juices; fruit juices; fizzy drinks; meals or snacks such as burgers, pizza, chicken, and fries; sweets and cakes; and crisps and other salty snacks. All participants were asked to record their habitual intake frequency for each food group within the last year according to the following categories:

1—never;
2—less often than once a month;
3—from one to three times a month;
4—once a week;
5—several times a week;
6—once a day;
7—several times a day.

### 2.4. Physical Activity and Sedentary Behaviors

Self-reported physical activity was recorded in the questionnaire on a scale ranging from 1—‘low’ and 2—‘moderate’ to 3 –‘high’. The description of the scale was presented separately for the physical activity in leisure and work/school time. For leisure time, ‘low’ means ‘sedentary lifestyle, watching TV, reading the press, books, light housework, taking a walk for 1–2 h a week’; ‘moderate’—‘walks, cycling, gymnastics, gardening or other light physical activity performed for 2–3 h a week ‘; and ‘high’—‘cycling, running, working on a plot or garden, and other sports activities requiring physical effort, taking up more than 3 h a week’. ‘Low’ activity at work/school time was described as ‘over 70% of the time in a sitting position’, ‘moderate’ as ‘approximately 50% of the time in a sitting position and about 50% of time moving’, and ‘high’ as ‘about 70% of the time in motion or doing physical work associated with a lot of effort’ [50]. Sedentary behaviors included reading books and newspapers, watching TV, and using the computer. Frequency of watching TV and reading books and newspapers was recorded using seven-point scales ranging from 1—never, 2—less than once a month, 3—one to three times a month, 4—once a week, 5—two to six times/week, and 6—once a day to 7—more than once a day. Using the computer was recorded on a six-point scale ranging from 1—never, 2—less than 1 h a day, 3—from 1 till almost 2 h a day, 4—from 2 till almost 4 h a day, and 5—from 4 till almost 6 h a day to 6—more than 6 h a day.

### 2.5. Socio-Demographic Variables

The questionnaire also collected information about sociodemographic characteristics of the sample: gender, age, education, and place of residence. Body Mass Index (BMI) was calculated using self-reported weight and height and categorized according to International Obesity Task Force (IOTF) standards [51].

### 2.6. Statistical Analysis

A principal components factor analysis (PCA) was conducted to derive dietary patterns based on the frequency of eating of sixteen food groups. The factors were rotated by an orthogonal (Varimax) transformation. The number of factors was based on the following criteria: components with an eigenvalue of 1, a scree plot test, and the interpretability of the factors. The eigenvalues signify the amount of variance explained by each of the factors. Food items were considered to load on a factor if they had an absolute correlation of 0.5 with it. The factorability of data was confirmed with the Kaiser-Meyer-Olkin (KMO) measure of sampling adequacy and Bartlett’s test of sphericity achieving statistical significance. The KMO value was 0.781, which attests the correct choice of analysis and the number of factors. Bartlett’s test had a significance of *p* < 0.0001 [52]. Five dietary patterns (factors) were derived: ‘Fruit & vegetables’ (comprising fruits and vegetables), ‘Wholemeal food’ (comprising wholemeal pasta, groats, and wholemeal bread), ‘Fast foods & sweets’ (comprising crisps and other salty snacks; meals or snacks such as burgers, pizza, chicken, and fries; sweets and cakes; and fizzy drinks), ‘Fruit & vegetable juices’ (comprising vegetable juices and fruit juices), and ‘Meat & meat products’ (comprising red meat, white meat, cured meats and sausages, and fried foods), accounting for 66.2% of total variance.

Based on the tertiles distribution, participants were divided into three categories within each pattern (bottom, middle, or upper tertile). The upper tertile (T3) represents the greatest adherence and the bottom tertile (T1) represents the lowest adherence to the DPs.

Logistic regression analysis was used to verify associations between variables describing physical activity and sedentary behaviors (independent variables), and DPs (dependent variables). The variables describing physical activity were as follows: moderate or high physical activity at work; moderate or high physical activity at leisure time; watching TV at least once a day, using the computer at least four hours a day; and reading books/newspapers at least once a day.

Odds ratios (ORs) represented the chances of adherence to the upper tertiles of each DP. The reference groups (OR = 1.00) were those that represented the bottom tertile of each DP. Wald’s test was used to assess the significance of ORs. Tests of a linear trend across increasing tertiles of DPs adherence (for ORs) were calculated for physical activity. *P*-value ≤ 0.05 was considered as significant for all tests. All analyses were carried out by applying sample weights to adjust for non-response and missing data. All analyses were performed using SAS 9.4. Software (SAS Institute Inc., Cary, NC, USA).

## 3. Results

### 3.1. Sample Characteristics

The sample consisted of 1007 participants (529 women and 478 men) aged 21 to 65 years. Table 1 displays the socio-demographic characteristics of the study sample.

### 3.2. Dietary Patterns

Table 2 illustrates the correlations between the frequency of eating of particular food groups and each of the five DPs.

The mean standardized values in individual tertiles are significantly different within DPs (Table 3).

### 3.3. Physical Activity and Sedentary Behaviors

**Figure 1:**
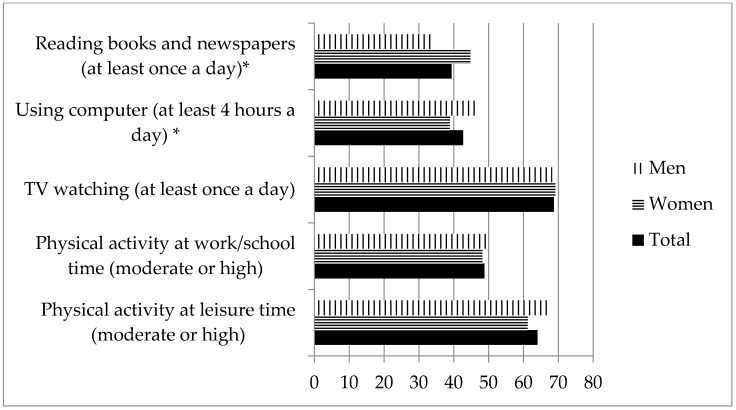
Self-reported physical activity and sedentary behaviors in the total sample, and in women and men separately. * Significantly differed according to gender (*p*-value ≤ 0.05, Chi-square test).

Almost 70% of the respondents watched TV at least once a day. Among them, 7.9% declared watching TV for less than one hour per day, 34.4%—from 1 to almost 2 h, 36.6%—from 2 to 4 h, 14.6%—from 4 to almost 6 h, and 6.5% watched TV for more than six hours per day. About 2/3 of the sample informed about moderate or high activity in leisure time, while such activity was indicated by less than 50% of respondents during work or school days. Slightly more than 2/5 of respondents used the computer every day for at least 4 h a day. Significantly more men than women were characterized by performing such activity. About 2/5 of respondents reported daily reading of books and newspapers, with significantly more women than men reporting such activity (Figure 1).

The correlations between self-reported physical activity, sedentary behaviors, and gender ranged from 0.106 to 0.396 (Pearson’s correlation coefficient) for significant positive correlations and from 0.081 to –0.154 for significant negative correlations (Table 4).

Gender correlated negatively with reading books/newspapers and using the computer. Watching TV correlated positively with reading books/newspapers and using the computer, and negatively with physical activity during leisure time. Using the computer correlated negatively with physical activity at work/school and during leisure time. The positive correlation between physical activity at work/school and during leisure time was one of the strongest (*r* = 0.364).

### 3.4. Dietary Patterns Versus Physical Activity

The results have demonstrated that people who consumed fruit and vegetables juices most often (the upper tertile of F&VJ) were more likely to display moderate or high physical activity at work/school. Also, people in the upper tertile of the ‘Wholemeal food’ pattern were more likely to exhibit moderate and high activity at work/school. However, those who ate fruit and vegetables most often (the upper tertile of F&V) were less likely to display moderate or high physical activity at work/school (Table 5).

Both women and men were more likely to exhibit moderate or high activity during work/school time in the upper tertile of the ‘Fruit & vegetable juices’ pattern. Only men who ate wholemeal food most often (the upper tertile of WF) were more likely to display at least moderate physical activity (Table 6 and Table 7).

Respondents in the upper tertiles of the ‘Wholemeal food’ and ‘Fruit & vegetable juices’ pattern were more likely to display moderate or high physical activity in leisure time. Both women and men in the upper tertiles of these DPs were more likely to display at least moderate physical activity in leisure time. In both dietary patterns, men were more likely to display moderate or high physical activity in leisure time (Table 6 and Table 7).

In the upper tertile of the ‘Fast food & sweets’ pattern, people were less likely to read books/newspapers at least once a day. The chances of such activity decreased by 50% amongst women in the upper tertile of this pattern. There was no significant association observed in the group of men. Respondents who most often consumed fruit and vegetables (the upper tertile of F&V), wholemeal food (the upper teritile of WF), and fruit and vegetable juices (the upper tertile of F&VJ) were more likely to read books/newspapers at least once a day. Both women and men were more likely to read books/newspapers at least once a day if they were in the upper tertiles of the F&V and WF patterns. Only women who consumed fruit and vegetable juices most often (the upper tertile of F&VJ) were more likely to read books/newspapers at least once a day.

People in the upper tertiles of the ‘Meat & meat products’ and the ‘Fruit & vegetable’ pattern were more likely to watch TV at least once a day. Both men and women were more likely to watch TV at least once a day in the upper tertile of M&MP. In men, watching TV at least once a day was 2.5 times more likely in the upper tertile of the ‘Fruit & vegetable’ pattern, while amongst women, there were no significant differences.

People in the upper tertile of the ‘Wholemeal food’ pattern were less likely to use the computer at least four hours a day. For women, using the computer at least four hours a day was less likely in the upper tertile of this pattern, while in the group of men, there were no significant differences (Table 5, Table 6 and Table 7).

## 4. Discussion

The frequent occurrence of sedentary behaviors is usually accompanied by lower physical activity, which is confirmed by both the direct measurement with the use of accelerometers [53,54] and the self-reported data [55]. In our study, weak negative associations were found between watching TV and using the computer, and self-reported physical activity, both during work/school and leisure time. Some previous studies have shown that the correlations between watching television and different physical activity domains (leisure, occupational, and total) were non-significant [1,37,56]. Similarly to other studies [53,57], we have found that moderate and high physical activity was related to a lower incidence of sedentary behaviors. However, we did not find any association between reading books/newspapers and self-reported physical activity. As in other studies, a positive association was found between watching TV and using the computer. Increasing the time spent in front of the TV and computer is a symptom of the lifestyle of a part of the Polish population. On the one hand, there is a growing group of people engaging in physical activity, while on the other hand, there are people with low physical activity who revealed many sedentary behaviors [58].

Watching TV and using the computer not only reduces energy expenditure, but can also lead to increasing the amount of food consumed, as demonstrated especially in the case of television. Spending time in front of the TV has been shown to coincide with additional calories intake during viewing [21,22,23]. While watching TV, one is exposed to an increasing number of advertisements for food high in fat, sugar, and salt [24,25], which is conducive to its consumption. Several studies have documented the increased intake of snack foods and energy, especially among adolescents who are more involved in watching TV [21,22,26,27]. In our study, there was no relation between watching television and the ‘Fast food & sweets’ pattern. Moreover, being in the upper tertile of this DP decreased the chance of reading books/newspapers at least once a day. These findings suggest that particular sedentary behaviors can be associated with specific eating behaviors.

Sedentary behaviors expend very little energy and similarly to low physical activity, were found to be associated with the consumption of unhealthy foods, especially in youth [10,21,26,27,59,60]. However, in our study, sedentary behaviors of adults (watching TV, using the computer, reading books or magazines) were found to be associated with the consumption of both unhealthy and healthy foods.

Representing unhealthy DP (‘Meat & meat products’) significantly increased the chance of watching TV at least once a day. Unlike in other studies [27], our study did not show an association between watching TV and ‘Fast food & sweets’ pattern. The lack of association between the consumption of certain food products and watching television in the countries of Central and Eastern Europe was also demonstrated in an earlier study [61]. In the Polish population, there is a high consumption of sweets regardless of the circumstances of their eating [62], which may explain the lack of association between watching TV and eating sweets.

Findings that confirmed an association between unhealthy dietary patterns and sedentary behaviors may help explain, at least in part, the relationship between sedentary behaviors and body-weight gain during adulthood [30,63,64]. A positive relationship between the ‘Meat & meat products’ pattern and TV viewing may seem unhelpful in explaining the increase in overweight and obese people. However, this pattern largely reflects the features of the traditional diet in Poland, named by Wadolowska et al. [45] as ‘traditional Polish’, which is characterized by large proportions of meat and a high caloric value.

In our study, healthy DPs were more likely to coincide with behaviors such as watching TV and reading books/newspapers. The chances of watching TV at least once a day significantly increased in the upper tertile of the ‘Fruit & vegetable’ pattern. In the study of Charreire et al. [37], an association between the ‘healthy food’ pattern and watching TV was not observed. The observed association between watching TV and reduced likelihood of regularly consuming fruit and vegetables [61] could be a part of a less healthy lifestyle or possibly a result of the replacement of fruit and vegetables by other foods advertised on TV more frequently. Although in Poland, the advertising of sweets and snacks is common in mass media, no negative association has been observed in our study. Therefore, the reason for the positive association between television viewing and the consumption of fruit and vegetables is not related to the emission of advertising, but probably results from the promotion of a more healthy lifestyle on TV. Higher chances of reading books/newspapers at least once a day were found in the upper tertiles of the healthy DPs (F&V, WF, and F&VJ). This sedentary behavior does not increase the energy expenditure, but it can be a source of knowledge about healthy eating behaviors.

Belonging to the upper tertiles of healthy DPs was associated with higher physical activity, with the exception of the ‘Fruit & vegetable’ pattern. People in the upper tertile of F&V were less likely to report moderate or high activity during work/school time. The latter result was not confirmed in other studies, which only demonstrated positive associations between the ‘healthy food’ patterns and leisure-time physical activity [65,66]. In adults, a high level of leisure time physical activity was found to be associated with a high consumption of healthy foods such as fruits (in men) and vegetables (both in men and women) [37]. Positive associations between leisure-time physical activity and intakes of specific macronutrients or healthy food groups have also been reported [38,67,68,69,70]. The Multiethnic Cohort Study found that physical activity (three times/week) was positively associated with the vegetable and fruit and milk patterns [71]. Similarly to Park et al. [71], we did not find any significant associations between self-reported physical activity and unhealthy DPs. By contrast, in the middle-aged French population, a negative association between an ‘Alcohol and meat’ pattern and physical activity was found [36].

Similarly to other studies [4,37,72,73,74], our results have demonstrated that associations between DPs and sedentary behaviors differed within gender. Women in the upper tertile of the ‘Wholemeal food’ pattern were less likely to use the computer for at least four hours a day. Women in the upper tertile of the ‘Fruit and vegetables juices’ pattern were more likely to read books/newspapers at least once a day, while the chances of reading were reduced in the upper tertile of the ‘Fast food & sweets’ pattern. Similar associations were not observed within men. Both women and men were more likely to read books/newspapers when they represented the ‘Wholemeal food’ pattern, as well as the ‘Fruit & vegetable’ pattern. The EPIC–Potsdam study found that physical activity level was positively associated with the ‘Fruit and vegetable’ dietary pattern in men and was negatively associated with the ‘Bread and sausage’ pattern in women [72]. In men, the ‘Alcohol/meat’ pattern was negatively associated with leisure-time physical activity and was not associated with occupational physical activity [37]. Fung et al. [17] also reported that a dietary pattern characterized by higher intakes of red meat, high-fat dairy products, beer, and liquor (‘Western pattern’) in men was associated with less leisure-time physical activity. We did not find any significant association between the ‘Meat & meat products’ pattern and self-reported physical activity, also among men. Nevertheless, in the upper tertile of this pattern, men were more likely to watch TV at least once a day.

In our study, sedentary behaviors were found to be associated with both unhealthy and healthy dietary patterns. This may be explained using models describing the determinants of behaviors change. Healthy lifestyles emphasize that an individual’s behavior is conducted to minimize health problems and to maximize their own well-being [75]. Individuals who engage in a healthy lifestyle change their behaviors in different ways, e.g., by increasing their intake of vegetables and fruits, their physical activity, sleeping time etc. Due to the Transtheoretical Model, at the given time, the change may be in one of five stages (precontemplation, contemplation, preparation, action, and maintenance) [76]. Moreover, changes in each of the spheres of life (diet, sleep, and physical activity) can occur at different times and with varying intensity, which may explain the simultaneous occurrence of healthy dietary habits and low physical activity or the inverse relationship. The occurrence of ’healthy vs unhealthy’ dietary behaviors together with sedentary behaviors can also be explained on the ground of the Health Belief Model. This model suggests that beliefs about health problems, perceived benefits of action and barriers to action, and self-efficacy explain people’s engagement in health-promoting behavior [76]. These factors can condition the changes of behaviors to a different extent. Moreover, in the socioecological model, one may find other reasons behind healthy vs unhealthy behaviors within an individual’s lifestyle. This model accounts for multiple factors (intrapersonal factors, interpersonal processes and primary groups, institutional or organizational factors, community factors, and public policies) that can influence the behavior change process in a different way [77]. Similarly, in the Social Cognitive Theory, it is indicated that personal, environmental, and behavioral factors have an influence on an individual’s ability to control lifestyle modification [78,79]. Thus, lifestyle being a dynamic process, one can observe the coexistence of healthy and unhealthy behaviors due to the existence of different stages in behavior change and many factors interacting with varying strength.

The observed links between both healthy as well as unhealthy DPs and some sedentary behaviors are of great importance for public health in Poland. In particular, the co-existence of healthy DPs and sedentary behaviors should be focused on when new pro-health initiatives are developed, e.g., the promotion of reading and healthy snacking. The strength of our results is that they are based on a relatively large representative sample of the Polish population. Nevertheless, the findings have some limitations. One of them relates to the potential biases that may occur when self-reported data is analyzed. The use of the questionnaire is also limited due to the overestimation of some foods’ consumption when FFQs is used. We have chosen FFQ because we aimed to see predominantly ‘healthy’ and ‘unhealthy’ dietary patterns, rather than an exact amount of foods. Moreover, the ideal measurements of physical activity would be a direct measure recorded by an accelerometer [80]. However, it was not logistically and economically possible in this study, taking into account the number of respondents. Although our findings should not be generalized to the population with different cultural backgrounds, our study provides an interesting insight into dietary patterns and their association with sedentary behaviors and the self-assessment of physical activity. The results can be used in the preparation of interventions targeted at health-related changes in lifestyle.

## 5. Conclusions

The study revealed the coexistence of the associations between both healthy as well as unhealthy dietary patterns and some sedentary behaviors. Frequent consumption of fruits and vegetables was conducive to watching TV and reading books/newspapers at least once a day. However, there was no such association in the case of the frequent consumption of fast foods, so newspapers and books cannot be recommended as carriers of educational messages encouraging to change such behaviors. The ‘Meat & meat products’ pattern, which best reflects the characteristics of the traditional Polish diet, coexists with greater chances of watching television frequently. This denotes the overlapping of negative lifestyle effects on the development of overweight and obesity.

Healthy DPs were more likely to coexist with self-reported moderate and high physical activity during work/school and leisure time, with the exception of the ‘Fruit & vegetables’ pattern, whereas significant associations were not demonstrated for unhealthy DPs. On one hand, this does not equate to the overlapping of negative consequences of unhealthy eating behavior and low physical activity, but on the other hand, association between the ‘Meat & meat products’ pattern and watching TV indicates the need to introduce changes not only in eating behaviors, but also in regard to sedentary behaviors.

## Figures and Tables

**Table 1:** Study sample characteristics.

Variables		*N* = 1007	%
Gender	Female	529	52.5
	Male	478	47.5
Age	21–34 years	370	36.7
	35–44 years	235	23.3
	45–54 years	132	13.2
	55–65 years	270	26.8
Place of residence	City ≤ 50,000 residents	539	53.5
	City > 50,000 residents	199	19.8
	Rural area	269	26.7
Education	Upper secondary and lower	403	40.1
	Higher	604	59.9

N—number of participants.

**Table 2:** Factor-loading matrix for the DPs identified by principal component analysis (PCA).

Variables	Factor 1 Fast Foods & Sweets	Factor 2 Meat & Meat Products	Factor 3 Fruit & Vegetable	Factor 4 Wholemeal Food	Factor 5 Fruit & Vegetable Juices
Crisps and other salty snacks	0.824				
Meals or snacks such as burgers, pizza, chicken, fries	0.756				
Sweets and cakes	0.702				
Fizzy drinks	0.633				
Red meat (pork, beef, venison)		0.783			
White meat (poultry, turkey)		0.748			
Cured meats and sausages		0.696			
Fried foods		0.551			
Fruits			0.825		
Vegetables			0.764		
Cheeses (including melted cheese, blue cheese)					
Wholemeal pasta, groats				0.839	
Wholemeal bread				0.763	
Fermented milk drinks					
Vegetable juices					0.830
Fruit juices					0.799
Variance Explained (%)	24.9	16.0	9.5	7.4	6.4
Total Variance Explained (%)	64.2				
Kaiser’s Measure of Sampling Adequacy:	0.781				

Factor loadings of ≤ |0.50| are not shown in the Table for simplicity.

**Table 3:** The mean standardized values in individual tertiles within DPs.

	Bottom Tertile (T1)		Middle Tertile (T2)		Upper Tertile (T3)		*p*-Value
	Mean	*N*	Mean	*N*	Mean	*N*	
Fast foods & sweets	0.323 ^a^	344	0.478 ^b^	325	0.639 ^c^	338	<0.001
Meat & meat products	0.506 ^a^	329	0.663 ^b^	340	0.776 ^c^	338	<0.001
Fruits & vegetables	0.438 ^a^	337	0.620 ^b^	340	0.781 ^c^	330	<0.001
Wholemeal food	0.351 ^a^	331	0.541 ^b^	355	0.681 ^c^	321	<0.001
Fruit & vegetable juices	0.288 ^a^	332	0.491 ^b^	353	0.682 ^c^	322	<0.001

N—number of participants; ^a,b,c^ Values with different letters in a row are significantly different across the tertiles (Waller-Duncan test).

**Table 4:** Person’s correlation coefficients for self-reported physical activity, sedentary behaviors, and gender.

Variables	(1)	(2)	(3)	(4)	(5)
Gender	−0.011	−0.116 **	−0.081 *	0.012	0.059
Watching TV (1)	1	0.106 **	0.396 **	−0.015	−0.101 **
Reading books and newspapers (2)		1	0.229 **	0.002	0.022
Using the computer (3)			1	−0.095 **	−0.091 **
Physical activity at work/school (4)				1	0.364 **
Physical activity during leisure time (5)					1

Statistically significant: * *p* < 0.05, ** *p* < 0.01.

**Table 5:** Adjusted associations between dietary patterns and activity components of lifestyle in the total sample (Adjusted Odds Ratios with 95% Confidence Intervals).

Variables	Fast Foods & Sweets (Ref. Bottom Tertile)		Meat & Meat Products (Ref. Bottom Tertile)		Fruit & Vegetable (Ref. Bottom Tertile)		Wholemeal Food (Ref. Bottom Tertile)		Fruit &Vegetable Juices (Ref. Bottom Tertile)	
	Upper Tertile	*p*	Upper Tertile	*p*	Upper Tertile	*p*	Upper Tertile	*p*	Upper Tertile	*p*
Physical activity at work/school time (moderate or high)	0.97 (0.71; 1.31)	0.836	0.95 (0.69; 1.29)	0.744	0.73 (0.53; 0.99)	0.043	1.46 (1.06; 2.00)	0.018	2.17 (1.58; 2.98)	<0.0001
Physical activity during leisure time (moderate or high)	0.96 (0.69; 1.32)	0.793	1.15 (0.82; 1.58)	0.418	1.18 (0.84; 1.63)	0.328	2.34 (1.67; 3.27)	<0.0001	1.93 (1.38; 2.68)	<0.001
Watching TV (at least once a day)	1.23 (0.88; 1.72)	0.215	1.73 (1.24; 2.39)	0.001	1.71 (1.22; 2.38)	0.002	0.91 (0.64; 1.27)	0.569	0.76 (0.53; 1.06)	0.113
Using computer (at least 4 hours a day)	0.84 0.62;1.15)	0.282	0.97 (0.71; 1.32)	0.858	1.18 (0.87; 1.62)	0.277	0.73 (0.53; 0.99)	0.043	1.19 (0.87; 1.62)	0.277
Reading books and newspapers (at least once a day)	0.60 (0.43; 0.83)	0.002	1.16 (0.83; 1.60)	0.386	2.77 (1.98; 3.86)	<0.0001	2.49 (1.77; 3.49)	<0.0001	1.54 (1.11; 2.14)	0.009

**Table 6:** Adjusted associations between dietary patterns and activity components of lifestyle in the sample of women (Adjusted Odds Ratios with 95% Confidence Intervals).

Variables	Fast Foods & Sweets (Ref. Bottom Tertile)		Meat & Meat Products (Ref. Bottom Tertile)		Fruit & Vegetable (Ref. Bottom Tertile)		Wholemeal Food (Ref. Bottom Tertile)		Fruit & Vegetable Juices (Ref. Bottom Tertile)	
	Upper Tertile	*p*	Upper Tertile	*p*	Upper Tertile	*p*	Upper Tertile	*p*	Upper Tertile	*p*
Physical activity at work/school time (moderate or high)	0.82 (0.53; 1.25)	0.362	1.09 (0.69; 1.69)	0.705	0.71 (0.45; 1.09)	0.118	1.34 (0.86; 2.07)	0.189	2.36 (1.52; 3.63)	<0.001
Physical activity during leisure time (moderate or high)	0.71 (0.45; 1.10)	0.131	1.33 (0.83; 2.12)	0.229	1.21 (0.76; 1.90)	0.414	2.43 (1.54; 3.83)	<0.001	1.65 (1.04; 2.60)	0.032
Watching TV (at least once a day)	1.39 (0.87; 2.21)	0.165	1.62 (1.01; 2.58)	0.042	1.25 (0.78; 1.99)	0.356	0.81 (0.49; 1.30)	0.3815	0.78 (0.48; 1.24)	0.303
Using Computer (at least 4 hours a day)	0.80 (0.52; 1.23)	0.319	0.99 (0.63; 1.55)	0.984	0.99 (0.63; 1.53)	0.964	0.64 (0.41; 0.98)	0.047	1.21 (0.78; 1.87)	0.390
Reading books and newspapers (at least once a day)	0.53 (0.34; 0.82)	0.005	1.04 (0.65; 1.64)	0.874	2.93 (1.84; 4.64)	<.0001	2.22 (1.40; 3.50)	<0.001	1.65 (1.05; 2.57)	0.027

**Table 7:** Adjusted associations between dietary patterns and activity components of lifestyle in the sample of men (Adjusted Odds Ratios with 95% Confidence Intervals).

Variables	Fast Foods & Sweets (Ref. Bottom Tertile)		Meat & Meat Products (Ref. Bottom Tertile)		Fruit & Vegetable (Ref. Bottom Tertile)		Wholemeal Food (Ref. Bottom Tertile)		Fruit & Vegetable Juices (Ref. Bottom Tertile)	
	Upper Tertile	*p*	Upper Tertile	*p*	Upper Tertile	*p*	Upper Tertile	*p*	Upper Tertile	*p*
Physical activity at work/school time (moderate or high)	1.10 (0.69; 1.75)	0.689	0.86 (0.54; 1.35)	0.505	0.79 (0.49; 1.26)	0.319	1.64 (1.02; 2.63)	0.039	1.99 (1.25; 3.18)	0.004
Physical activity during leisure time (moderate or high)	1.15 (0.69; 1.89)	0.589	0.95 (0.58; 1.55)	0.838	1.56 (0.91; 2.66)	0.102	2.70 (1.58; 4.58)	<0.001	2.27 (1.38; 3.72)	0.001
Watching TV (at least once a day)	1.09 (0.66; 1.79)	0.738	1.65 (1.02; 2.67)	0.041	2.52 (1.47; 4.29)	<0.001	0.99 (0.59; 1.63)	0.955	0.74 (0.44; 1.24)	0.259
Using computer (at least 4 hours a day)	0.98 (0.62; 1.56)	0.959	1.02 (0.65; 1.61)	0.902	1.31 (0.82; 2.10)	0.249	0.76 (0.48; 1.21)	0.260	1.20 (0.76; 1.90)	0.429
Reading books and newspapers (at least once a day)	0.75 (0.45; 1.22)	0.244	1.39 (0.84; 2.27)	0.194	2.32 (1.39; 3.84)	<0.001	2.59 (1.55; 4.30)	<0.001	1.49 (0.90; 2.44)	0.114

## References

[1] Biddle S.J. (2007). Sedentary behavior. Am. J. Prev. Med..

[2] Dunstan D.W., Salmon J., Owen N., Armstrong T., Zimmet P.Z., Welborn T.A., Cameron A.J., Dwyer T., Jolley D., Shaw J.E. (2005). Associations of TV viewing and physical activity with the metabolic syndrome in Australian adults. Diabetologia.

[3] Hu F.B., Li T.Y., Colditz G.A., Willett W.C., Manson J.E. (2003). Television watching and other sedentary behaviors in relation to risk of obesity and type 2 diabetes mellitus in women. JAMA.

[4] Nurwanti E., Uddin M., Chang J.-S., Hadi H., Syed-Abdul S., Su E.C.-Y., Nursetyo A.A., Bhuiyan Masud J.H., Bai C.-H. (2018). Roles of Sedentary Behaviors and Unhealthy Foods in Increasing the Obesity Risk in Adult Men and Women: A Cross-Sectional National Study. Nutrients.

[5] Warburton D.E.R., Nicol C.W., Bredin S.S.D. (2006). Health benefits of physical activity: The evidence. CMAJ.

[6] Myers J., Kaykha A., George S., Abella J., Zaheer N., Lear S., Yamazaki T., Froelicher V. (2004). Fitness versus physical activity patterns in predicting mortality in men. Am. J. Med..

[7] Telama R., Yang X. (2000). Decline of physical activity from youth to young adulthood in Finland. Med. Sci. Sports Exerc..

[8] Jans M.P., Proper K.I., Hildebrandt V.H. (2007). Sedentary behavior in Dutch workers: Differences between occupations and business sectors. Am. J. Prev. Med..

[9] Lowry R., Wechsler H., Galuska D.A., Fulton J.E., Kann L. (2002). Television viewing and its associations with overweight, sedentary lifestyle, and insufficient consumption of fruits and vegetables among US high school students: Differences by race, ethnicity, and gender. J. Sch. Health.

[10] Rosenberg D.E., Norman G.J., Sallis J.F., Calfas K.J., Patrick K. (2007). Covariation of adolescent physical activity and dietary behaviors over 12 months. J. Adolesc. Health.

[11] Rehm C.D., Matte T.D., Van Wye G., Young C., Frieden T.R. (2008). Demographic and behavioral factors associated with daily sugar-sweetened soda consumption in New York City adults. J. Urban Health.

[12] Hobbs M., Pearson N., Foster P.J., Biddle S.J. (2014). Sedentary behaviour and diet across the lifespan: An updated systematic review. Br. J. Sports Med..

[13] Compernolle S., De Cocker K., Teixeira P.J., Oppert J.-M., Roda C., Mackenbach J.D., Lakerveld J., McKee M., Glonti K., Rutter H. (2016). The associations between domain-specific sedentary behaviours and dietary habits in European adults: A cross-sectional analysis of the SPOTLIGHT survey. BMC Public Health.

[14] Hamilton M.T., Hamilton D.G., Zderic T.W. (2007). Role of low energy expenditure and sitting in obesity, metabolic syndrome, type 2 diabetes, and cardiovascular disease. Diabetes.

[15] De Rezende L.F.M., Lopes M.R., Rey-López J.P., Matsudo V.K.R., Do Carmo Luiz O. (2014). Sedentary behavior and health outcomes: An overview of systematic reviews. PLoS ONE.

[16] Pearson N., Biddle S.J. (2011). Sedentary behavior and dietary intake in children, adolescents, and adults: A systematic review. Am. J. Prev. Med..

[17] Fung T.T., Hu F.B., Yu J., Chu N.F., Spiegelman D., Tofler G.H., Willett W.C., Rimm E.B. (2000). Leisure-time physical activity, television watching, and plasma biomarkers of obesity and cardiovascular disease risk. Am. J. Epidemiol..

[18] Fletcher E., Leech R., McNaughton S.A., Dunstan D.W., Lacy K.E., Salmon J. (2015). Is the relationship between sedentary behaviour and cardiometabolic health in adolescents independent of dietary intake? A systematic review. Obes. Rev..

[19] Cureau F.V., Ekelund U., Bloch K.V., Schaan B.D. (2017). Does body mass index modify the association between physical activity and screen time with cardiometabolic risk factors in adolescents? Findings from a country-wide survey. Int. J. Obes..

[20] Thorp A.A., McNaughton S.A., Owen N., Dunstan D.W. (2013). Independent and joint associations of TV viewing time and snack food consumption with the metabolic syndrome and its components; a cross-sectional study in Australian adults. Int. J. Behav. Nutr. Phys. Act..

[21] Van Den Bulck J., Van Mierlo J. (2004). Energy intake associated with television viewing in adolescents, a cross sectional study. Appetite.

[22] Scully M., Dixon H., Wakefield M. (2009). Association between commercial television exposure and fast-food consumption among adults. Public Health Nutr..

[23] Bowman S.A. (2006). Television-viewing characteristics of adults: Correlations to eating practices and overweight and health status. Prev. Chronic Dis..

[24] Coon K.A., Goldberg J., Rogers B.L., Tucker K.L. (2001). Relationships between use of television during meals and children’s food consumption patterns. Pediatrics.

[25] Hastings G., Stead M., McDermott L., Forsyth A., MacKintosh A.M., Rayner M., Godfrey C., Caraher M., Angus K. (2003). Review of Research on the Effects of Food Promotion to Children [Online].

[26] Utter J., Neumark-Sztainer D., Jeffery R., Story M. (2003). Couch potatoes or French fries: Are sedentary behaviors associated with body mass index, physical activity, and dietary behaviors among adolescents?. J. Am. Diet. Assoc..

[27] Stroebele N., de Castro J.M. (2004). Television viewing is associated with an increase in meal frequency in humans. Appetite.

[28] Ruano C., Henriquez P., Martínez-González M.Á., Bes-Rastrollo M., Ruiz-Canela M., Sánchez-Villegas A. (2013). Empirically derived dietary patterns and health-related quality of life in the SUN project. PLoS ONE.

[29] Thorpe M.G., Milte C.M., Crawford D., McNaughton S.A. (2016). A comparison of the dietary patterns derived by principal component analysis and cluster analysis in older Australians. Int. J. Behav. Nutr. Phys. Act..

[30] Heerman W.J., Jackson N., Hargreaves M., Mulvaney S.A., Schlundt D., Wallston K.A., Rothman R.L. (2017). Clusters of healthy and unhealthy eating behaviors are associated with Body Mass Index among adults. J. Nutr. Educ. Behav..

[31] Smith A.D., Emmett P.M., Newby P.K., Northstone K. (2011). A comparison of dietary patterns derived by cluster and principal components analysis in a UK cohort of children. Eur. J. Clin. Nutr..

[32] Newby P.K., Tucker K.L. (2004). Empirically derived eating patterns using factor or cluster analysis: A review. Nutr. Rev..

[33] Gustaw-Rothenberg K. (2009). Dietary patterns associated with Alzheimer’s disease: Population based study. Int. J. Environ. Res. Public Health.

[34] Shin D., Lee K.W., Kim M.H., Kim H.J., An Y.S., Chung H.K. (2018). Identifying dietary patterns associated with mild cognitive impairment in older Korean adults using reduced rank regression. Int. J. Environ. Res. Public Health.

[35] Nanri A., Mizoue T., Shimazu T., Ishihara J., Takachi R., Noda M., Iso H., Sasazuki S., Sawada N., Tsugane S. (2017). Dietary patterns and all-cause, cancer, and cardiovascular disease mortality in Japanese men and women: The Japan public health center-based prospective study. PLoS ONE.

[36] Kesse-Guyot E., Bertrais S., Peneau S., Estaquio C., Dauchet L., Vergnaud A.-C., Czernichow S., Galan P., Hercberg S., Bellisle F. (2009). Dietary patterns and their sociodemographic and behavioural correlates in French middle-aged adults from the SU.VI.MAX cohort. Eur. J. Clin. Nutr..

[37] Charreire H., Kesse-Guyot E., Bertrai S., Simon C., Chaix B., Weber C., Touvier M., Galan P., Hercberg S., Oppert J.-M. (2011). Associations between dietary patterns, physical activity (leisure-time and occupational) and television viewing in middle-aged French adults. Br. J. Nutr..

[38] Cho E.R., Shin A., Lim S-Y., Kim J. (2010). Dietary patterns and their associations with health behaviours in Korea. Public Health Nutr..

[39] Esmaillzadeh A., Entezari M., Paknahad Z., Safavi M., Jalali M., Ghiasvand R. (2008). Identification of diet–disease relations through dietary pattern approach: A review. J. Res. Med. Sci..

[40] Vissers P.A.J., Jones A.P., van Sluijs E.M.F., Jennings A., Welch A., Cassidy A., Griffin S.J. (2013). Association between diet and physical activity and sedentary behaviours in 9–10-year-old British White children. Public Health.

[41] Wammes B., French S., Brug J. (2007). What young Dutch adults say they do to keep from gaining weight: Self-reported prevalence of overeating, compensatory behaviours and specific weight control behaviours. Public Health Nutr..

[42] Lee I., Djoussé L., Sesso H.D., Wang L., Buring J.E. (2010). Physical activity and weight gain prevention. JAMA.

[43] McAloney K., Graham H., Hall J., Law C., Platt L., Wardle H. (2012). OP13 Diet and physical activity levels among UK youth. J. Epidemiol. Community Health.

[44] Gaylis J.B., Levy S.S., Kviatkovsky S., DeHamer R., Hong M.Y. (2017). Relationships between physical activity, food choices, gender and BMI in Southern Californian teenagers. Int. J. Adolesc. Med. Health.

[45] Wadolowska L., Kowalkowska J., Lonnie M., Czarnocinska J., Jezewska-Zychowicz M., Babicz-Zielinska E. (2016). Associations between physical activity patterns and dietary patterns in a representative sample of Polish girls aged 13–21 years: A cross-sectional study (GEBaHealth Project). BMC Public Health.

[46] Leech R.M., McNaughton S.A., Timperio A. (2014). The clustering of diet, physical activity and sedentary behavior in children and adolescents: A review. Int. J. Behav. Nutr. Phys. Act..

[47] Ottevaere C., Huybrechs I., Benser J., De Bourdeaudhuij I., Cuenca-Garcia M., Dallongeville J., Zaccaria M., Gottrand F., Kersting M., Rey-López J.P. (2011). Clustering patterns of physical activity, sedentary and dietary behavior among European adolescents: The HELENA study. BMC Public Health.

[48] Wirfält A.K.E., Jeffery R.W. (1997). Using cluster analysis to examine dietary patterns: Nutrient intakes, gender, and weight status differ across food pattern clusters. J. Am. Diet. Assoc..

[49] Wirfält E., Drake I., Wallström P. (2013). What do review papers conclude about food and dietary patterns?. Food Nutr. Res..

[50] Beliefs and Eating Habits Questionnaire Behavioral Conditions of Nutrition Team, Committee of Human Nutrition Science. Polish Academy of Science. Warsaw 2014..

[51] Cole T.J., Lobstein T. (2012). Extended international (IOTF) body mass index cut-offs for thinness, overweight and obesity. Pediatr. Obes..

[52] Field A. (2009). Discovering Statistics Using SPSS.

[53] Nang E.E., Salim A., Wu Y., Tai E.S., Lee J., Van Dam R.M. (2013). Television screen time, but not computer use and reading time, is associated with cardio-metabolic biomarkers in a multiethnic Asian population: A cross-sectional study. Int. J. Behav. Nutr. Phys. Act..

[54] Kilani H., Al-Hazzaa H., Waly M.I., Musaiger A. (2013). Lifestyle Habits Diet, physical activity and sleep duration among Omani adolescents. Sultan Qaboos Univ. Med. J..

[55] Jefferis B.J., Sartini C., Ash S., Lennon L.T., Goya Wannamethee S., Whincup P.H. (2016). Validity of questionnaire-based assessment of sedentary behaviour and physical activity in a population-based cohort of older men; comparisons with objectively measured physical activity data. Int. J. Behav. Nutr. Phys. Act..

[56] Stamatakis E., Hirani V., Rennie K. (2009). Moderate-to-vigorous physical activity and sedentary behaviours in relation to body mass index-defined and waist circumference-defined obesity. Br. J. Nutr..

[57] Mansoubi M., Pearson N., Biddle S.J., Clemes S. (2014). The relationship between sedentary behaviour and physical activity in adults: A systematic review. Prev. Med..

[58] Nawrocka A., Mynarski A., Cholewa J., Garbaciak W. (2017). Leisure-time Physical Activity of Polish White-collar Workers: A Cross-sectional Study. Hong Kong J. Occup. Ther..

[59] Lioret S., Touvier M., Lafay L., Volatier J.L., Maire B. (2008). Dietary and physical activity patterns in French children are related to overweight and socioeconomic status. J. Nutr..

[60] Deshmukh-Taskar P.R., O’Neil C.E., Nicklas T.A., Yang S.-J., Liu Y., Gustat J., Berenson G.S. (2009). Dietary patterns associated with metabolic syndrome, sociodemographic and lifestyle factors in young adults: The Bogalusa Heart Study. Public Health Nutr..

[61] Vereecken C.A., Todd J., Roberts C., Mulvihill C., Maes L. (2005). Television viewing behaviour and associations with food habits in different countries. Public Health Nutr..

[62] Jeżewska-Zychowicz M., Jeznach M., Kosicka-Gębska M. (2013). Consumers’ interests in sweets with health-promoting properties and their selected determinants. Pol. J. Food Nutr. Sci..

[63] Cleland V.J., Schmidt M.D., Dwyer T., Venn A.J. (2008). Television viewing and abdominal obesity in young adults: Is the association mediated by food and beverage consumption during viewing time or reduced leisure-time physical activity?. Am. J. Clin. Nutr..

[64] Kant A.K. (2004). Dietary patterns and health outcomes. J. Am. Diet. Assoc..

[65] Olinto M.T.A., Willett W.C., Gigante D.P., Victora C.G. (2010). Sociodemographic and lifestyle characteristics in relation to dietary patterns among young Brazilian adults. Public Health Nutr..

[66] Kourlaba G., Panagiotakos D., Mihas K., Alevizos A. (2009). Dietary patterns in relation to socio-economic and lifestyle characteristics among Greek adolescents: A multivariate analysis. Public Health Nutr..

[67] Oppert J.M., Thomas F., Charles M.A., Benetos A., Basdevant A., Simon C. (2006). Leisure-time and occupational physical activity in relation to cardiovascular risk factors and eating habits in French adults. Public Health Nutr..

[68] Gillman M.W., Pinto B.M., Tennstedt S., Glanz K., Marcus B., Friedman R.H. (2001). Relationships of physical activity with dietary behaviors among adults. Prev. Med..

[69] Van Dam R.M., Grievink L., Ocke M.C., Feskens E.J. (2003). Patterns of food consumption and risk factors for cardiovascular disease in the general Dutch population. Am. J. Clin. Nutr..

[70] Chan R., Chan D., Woo J. (2012). Associations between dietary patterns and demographics, lifestyle, anthropometry and blood pressure in Chinese community-dwelling older men and women. J. Nutr. Sci..

[71] Park S.Y., Murphy S.P., Wilkens L.R., Yamamoto J.F., Sharma S., Hankin J.H., Henderson B.E., Kolonel L.N. (2005). Dietary patterns using the Food Guide Pyramid groups are associated with sociodemographic and lifestyle factors: The Multiethnic Cohort Study. J. Nutr..

[72] Bamia C., Orfanos P., Ferrari P., Overvad K., Hundborg H.H., Tjønneland A., Olsen A., Kesse E., Boutron-Ruault M.C., Clavel-Chapelon F. (2005). Dietary patterns among older Europeans: The EPIC–Elderly study. Br. J. Nutr..

[73] Holmbäck I., Ericson U., Gullberg B., Wirfält E. (2010). A high eating frequency is associated with an overall healthy lifestyle in middle-aged men and women and reduced likelihood of general and central obesity in men. Br. J. Nutr..

[74] Camoes M., Lopes C. (2008). Dietary intake and different types of physical activity: Full-day energy expenditure, occupational and leisure-time. Public Health Nutr..

[75] King D.E., Mainous A.G., Carnemolla M., Everett C.J. (2009). Adherence to healthy lifestyle habits in us adults, 1988–2006. Am. J. Med..

[76] Glanz K., Rimer B., Lewis F. (2002). Health Behavior and Health Education.

[77] McLeroy K.R., Bibeau D., Steckler A., Glanz K. (1988). An ecological perspective on health promotion programs. Health Educ. Behav..

[78] Bandura A. (1986). Social Foundations of thought and Action: A Social Cognitive Theory.

[79] Ihwanudin N.K., Amatayakul A., Karuncharernpanit S. (2015). Lifestyle modification effect on behavior change and physical conditions among hypertensive elderly in West Java, Indonesia. J. Health Res..

[80] Sylvia L.G., Bernstein E.E., Hubbard J.L., Keating L., Anderson E.J. (2014). Practical guide to measuring physical activity. J. Acad. Nutr. Diet..

